# Impact of filtering face pieces (FFP3) respiratory protective mask usage on the respiratory functions of informal sector carpenters in Douala: a five-month before-after study

**DOI:** 10.11604/pamj.2023.46.52.41225

**Published:** 2023-10-09

**Authors:** Catherine Bouland, Jean Junior Eye Ngoa, Jules Nebo, Joseph Francis Nde Djiele

**Affiliations:** 1 Center for Research in Environmental and Occupational Health, Environmental Health University of Brussels, School of Public Health, Route de Lennik, Brussels, Belgium; 2 Medical Practice, Field Investigating, Yaounde, Cameroon; 3 Cameroonian Society of General Practitioners, Yaounde, Cameroon

**Keywords:** Occupational, wood dust, workforce, lung, masks, protection, spirometry

## Abstract

**Introduction:**

informal sector carpenters in Douala, Cameroon, face potential risks to their respiratory health due to daily exposure to fine particles and wood dust. The study aims to demonstrate the importance of preventing respiratory problems in this population through regular use of filtering face pieces (FFP3) respiratory masks.

**Methods:**

the before-after study involved 37 carpenters who wore FFP3 masks during their professional activities for five months. Spirometry measurements were taken before and after the intervention to assess changes in respiratory function.

**Results:**

significant improvements were observed in forced vital capacity (FVC) 89.6 % to 95.0 % (p<0.000), forced expiratory volume in one second (FEV1) 88.1 % to 95.0 % (p<0.000), Tiffeneau index 82.4 to 84.9 (p<0.000), and peak expiratory flow (PEF) 6.7 l/s to 7.9 l/s (p<0.000) after mask usage, indicating enhanced lung function.

**Conclusion:**

the regular use of FFP3 masks had a positive impact on the respiratory health of informal sector carpenters in Douala, enhancing lung function and reducing airway obstruction. The study highlights the importance of preventive measures to safeguard the respiratory well-being of workers exposed to occupational hazards. Spell out Greek characters (i.e: alpha, beta).

## Introduction

Informal sector carpenters in Douala, Cameroon, face potential risks to their respiratory health due to their daily exposure to fine particles and wood dust in their work environment [1,2]. These precarious working conditions expose them to chronic respiratory problems, including asthma and bronchitis, which can impair their quality of life and ability to perform their trade [3,4]. In this context, the use of respiratory protective masks, such as FFP3 masks, has become a common practice in the industry. The informal carpentry sector presents inherent risks to the respiratory health of artisans, who are exposed daily to fine particles and aerosols during tasks such as cutting, sanding, and machine use. These work conditions can lead to persistent respiratory issues, indicating reduced lung function and general well-being. To assess whether preventive use of respiratory masks could improve their respiratory health, participants underwent spirometry measurements before and after the intervention, while continuing their carpentry activities. This before-after study, conducted over a period of five months from December 2019 to May 2020, aims to demonstrate the importance of preventing respiratory problems in informal sector carpenters through regular use of FFP3 respiratory masks. The study holds crucial significance in highlighting the beneficial effect of regular FFP3 mask-wearing for the prevention of respiratory problems among informal sector carpenters. The findings can further raise awareness about the importance of respiratory safety in this occupational domain and encourage the widespread adoption of similar preventive measures. By demonstrating the substantial advantages of using respiratory protective masks, this study aspires to enhance the health and well-being of these artisans while underscoring the paramount significance of prevention in preserving their long-term respiratory health. The research's scientific background and rationale lie in the need to address the occupational hazards faced by informal sector carpenters and evaluate the potential benefits of using FFP3 masks to safeguard their respiratory health. The study seeks to contribute valuable insights into the effectiveness of preventive measures in improving the lung function of this specific workforce and provide evidence-based support for respiratory protection in the informal sector. Objectives: i) To assess the impact of regular FFP3 respiratory mask usage on spirometric parameters, including forced vital capacity (FVC), forced expiratory volume in one second (FEV1), Tiffeneau index, and peak expiratory flow (PEF), among informal sector carpenters in Douala; ii) to compare the spirometric measurements before and after the five-month intervention period to evaluate any significant changes in respiratory function. Hypotheses: i) H0 (Null hypothesis): there will be no significant difference in spirometric parameters before and after the intervention, indicating that FFP3 mask usage has no impact on the respiratory functions of informal sector carpenters. ii) H1 (Alternative hypothesis): there will be a significant improvement in spirometric parameters after the five-month FFP3 mask intervention, indicating that regular mask usage positively influences the respiratory health of informal sector carpenters.

## Methods

**Study design:** the study design is a before-after study, also known as a pre-post intervention study. It involves assessing the respiratory functions of informal sector carpenters before and after a five-month intervention period during which they wear FFP3 respiratory masks while performing their professional activities. Spirometry measurements will be taken at two time points: before the intervention (baseline) and after the five-month intervention.

**Setting:** the study was conducted in Douala, Cameroon, which is a major city with a significant informal carpentry sector. Douala is known for its high levels of air pollution and occupational hazards related to woodworking activities. The locations of data collection include various carpentry workshops and sites within the city.

**Relevant dates:** the study was carried out over a period of five months, from December 2019 to May 2020. The recruitment of participants and baseline spirometry measurements took place in December 2019, before the intervention started. The intervention, which involved the regular usage of FFP3 masks, began in January 2020 and continued until May 2020. The follow-up spirometry measurements were conducted in May 2020, after the five-month intervention period was completed.

### Periods of recruitment, exposure, follow-up, and data collection

**Recruitment:** participants were recruited in December 2019. Informal sector carpenters in Douala who met the inclusion criteria were invited to participate in the study. The inclusion criteria involved being active carpenters, aged between 18 and 65 years, and having a minimum of one year of work experience in carpentry.

**Exposure:** the exposure period involved the five months from January 2020 to May 2020, during which the participants used FFP3 respiratory masks regularly while performing their carpentry tasks. They were provided with the masks and instructed to wear them throughout their working hours.

**Follow-up:** the follow-up period took place in May 2020, after the intervention ended. At this time, the participants underwent spirometry measurements again to evaluate any changes in their respiratory functions.

**Data collection:** data collection for spirometry measurements was conducted at two time points: baseline (December 2019) and follow-up (May 2020). The spirometry tests were performed by trained healthcare professionals following standardized procedures to ensure accurate and reliable results.

**Participants:** in this particular study, the participants are informal sector carpenters in Douala, Cameroon. The inclusion criteria involve being active carpenters, aged between 18 and 65 years, and having a minimum of one year of work experience in carpentry. The study does not involve comparing the outcomes of different groups; instead, it assesses changes in individual participants' respiratory functions before and after the five-month intervention period of using FFP3 respiratory masks. Each participant's baseline spirometry measurements were taken before the intervention (pre-intervention), and follow-up spirometry measurements were taken after the five-month intervention period (post-intervention). By comparing these measurements within each participant, the study evaluates the impact of regular FFP3 mask usage on their respiratory functions over time.

**Outcomes:** spirometric parameters: the primary outcomes of interest are spirometric parameters, including forced vital capacity (FVC), forced expiratory volume in one second (FEV1), Tiffeneau index (FEV1/FVC ratio), and peak expiratory flow (PEF). These measurements provide insights into the participants' respiratory function and lung health.

**Exposure:** filtering face pieces (FFP3) respiratory m usage: the main exposure of interest is the regular usage of FFP3 respiratory masks by the informal sector carpenters during their professional activities. The participants were provided with FFP3 masks and instructed to wear them throughout their working hours during the intervention period.

**Predictors:** duration of FFP3 mask usage: the duration of FFP3 mask usage, measured in months, is a predictor variable of interest. This variable represents the period of time the participants consistently wore the masks during their carpentry activities.

### Potential confounders

**Age:** age is a potential confounding factor as it can influence the participants' baseline respiratory functions and the effect of mask usage on lung health.

**Smoking status:** smoking status may affect the participants' respiratory health independently of the intervention and should be considered as a potential confounder.

**Previous respiratory conditions:** participants with pre-existing respiratory conditions may have different baseline respiratory functions, and their conditions could confound the impact of mask usage on lung health.

**Occupational exposure to other respiratory hazards:** the participants' exposure to other occupational respiratory hazards, apart from woodworking dust, may influence their respiratory health.

### Effect modifiers

**Gender:** gender may act as an effect modifier, as there might be differences in the impact of FFP3 mask usage on respiratory functions between males and females. Years of professional experience: The number of years the participants have worked as carpenters could potentially modify the effect of mask usage on their lung function.

**Diagnostic criteria:** there are no specific diagnostic criteria for the outcomes of spirometric parameters in this study. Spirometry is a standard procedure used to assess respiratory function, and the measurements are interpreted using reference values based on age, height, and gender to identify any abnormalities or changes in lung function. The pre-intervention spirometry measurements serve as baseline values for each participant, and the post-intervention measurements are used to compare changes in respiratory function over the study period. By clearly defining the outcomes, exposure, predictors, potential confounders, and effect modifiers, the study aims to conduct a comprehensive analysis to evaluate the impact of FFP3 mask usage on the respiratory functions of informal sector carpenters in Douala. This approach allows for accurate interpretation of the study's findings and minimizes bias to draw meaningful conclusions about the intervention's effectiveness. The data sources and measurement methods provided remain accurate for this before-after study design. The spirometric parameters, FFP3 mask usage, and potential confounders and effect modifiers were collected for each participant at two time points, ensuring internal comparability within the study population.

### Bias

**Efforts to address potential sources of bias**

**Selection bias:** to minimize selection bias, a systematic approach was used to recruit participants from the informal carpentry sector in Douala. Inclusion criteria, such as being active carpenters aged between 18 and 65 years with at least one year of work experience, were defined to ensure the study population represents the target group accurately. Moreover, efforts were made to include a diverse sample of carpenters from different workshops and sites to enhance the generalizability of the findings.

**Information bias:** to reduce information bias, spirometry measurements and other data were collected by trained healthcare professionals following standardized protocols. This helps ensure accurate and consistent data collection, reducing the likelihood of measurement errors or misclassification of exposure and outcome variables. Participants were also given clear instructions on self-reporting FFP3 mask usage to minimize reporting bias.

**Confounding:** in our study, we did not specifically address potential confounding effects, as the spirometer used already accounted for relevant covariates such as age and gender in its measurements. Additionally, with a sample size of 37, dividing participants into groups for further analysis could introduce bias in the statistical analysis. Furthermore, our study design involved comparing individuals to themselves before and after using the FFP3 mask, which mitigates the impact of confounding factors on the observed spirometric outcomes.

**Study size:** the study size was determined to ensure adequate statistical power to detect meaningful changes in spirometric parameters before and after the intervention. A sample size calculation was performed based on factors such as expected effect size, variability in spirometric measurements, significance level, and desired statistical power [5,6]. The calculated sample size required for the paired design was initially 31 subjects, but a target sample size of 310 subjects was chosen to account for potential “no-shows” and ensure sufficient participants for paired measurements. The larger sample size enhances the study's ability to detect significant differences and increases the generalizability of the findings to the target population, ensuring the study's statistical validity and meaningful conclusions.

**Quantitative variables handling in the analyses:** in our before-after study, the main quantitative variables of interest are the spirometric parameters, such as forced vital capacity (FVC), forced expiratory volume in one second (FEV1), Tiffeneau index (FEV1/FVC ratio), and peak expiratory flow (PEF). These quantitative variables were handled as follows in the analyses.

**Pre-intervention and post-intervention measurements:** for each participant, spirometry measurements were taken at two time points: before the intervention (baseline) and after the five-month intervention period (follow-up). The raw numerical values of FVC, FEV1, Tiffeneau index, and PEF were recorded for both time points.

**Comparison within participants:** as a before-after study, the primary analysis focused on comparing spirometric parameters within each participant. The change in spirometric measurements from baseline to follow-up was calculated for each participant. This was done by subtracting the baseline values from the follow-up values to obtain the change scores.

**Statistical analysis:** the change scores for each spirometric parameter were used as the outcome variables in the statistical analysis. Paired t-test was used to compare the mean changes in spirometric parameters before and after the intervention. This test allowed for the assessment of the significance of the changes and whether the regular usage of FFP3 masks had a significant impact on respiratory functions.

**Subgroup analyses:** we did not subdivide our sample for the reason above mentioned.

**Adjusting for confounding variables:** with this number, we didn´t adjust for potential confounding variables identified like age and smoking status as above mentioned. No regression models.

**Groupings of quantitative variables:** in our before-after study, there are no separate groups to compare; each participant serves as their own control. The primary analysis involves comparing the individual changes in spirometric parameters before and after the intervention for each participant. Therefore, there are no specific groupings chosen for quantitative variables in this study design. Instead, the focus is on assessing changes within each participant over time. By handling quantitative variables in this manner, the study can evaluate the impact of regular FFP3 mask usage on the respiratory functions of informal sector carpenters in Douala, providing valuable insights into the intervention's effectiveness in improving their lung health.

**Statistical methods for before-after study:** we used paired t-tests to compare the mean differences in the outcome variable (spirometric parameters) between the two time points within each participant. Paired t-tests are appropriate for normally distributed data and are commonly used in before-after studies to assess the intervention's impact.

**Descriptive statistics:** we used descriptive statistics to summarize the baseline characteristics of the study participants and the changes in the outcome variables after the intervention.

**Confidence intervals:** confidence intervals are calculated around the mean differences in the outcome variables before and after the intervention. These intervals provide a range within which the true population parameter is likely to lie with a certain level of confidence. In this before-after study design, the primary focus is on analyzing changes in outcomes within each participant, using paired t-tests to determine if the intervention had a significant effect on the respiratory functions of informal sector carpenters in Douala. Descriptive statistics, effect size calculations, and confidence intervals further provide valuable insights into the magnitude and significance of the observed changes.

**Sampling approach:** this study employed a combination of cluster sampling and convenience sampling to select participants from the city of Douala, which is divided into 6 administrative districts: Douala I, Douala II, Douala III, Douala IV, Douala V, and Douala VI. All six districts were selected for inclusion in the study.

**Sample size calculation:** the sample size was initially calculated to achieve a statistical power of 80%, a 95% confidence level, and a 5% significance level for a paired two-sample design. Based on the expected improvement in spirometric parameters (PEF, FVC, Tiffeneau index, and FEV1) from an average of 95% to 100% of their own expected values, the calculated sample size required for this study was 31 subjects based on the sample size formula for paired tests [5,6]. However, to account for potential “no-shows” due to the COVID crisis and ensure sufficient participants for the paired measurements, a tenfold margin was applied, leading to a target sample size of 310 subjects.

**Portable spirometer use:** the use of the portable spirometer in this study is justified due to its comparable results to a spirometer used in a specialized pulmonary center when used correctly [7]. It is crucial to ensure that the spirometer is operated accurately to obtain reliable and consistent data. The fact that both types of spirometers yield similar variations in results suggests that any discrepancies are more likely attributed to user error or subject cooperation rather than the method's reliability [8,9]. Moreover, the portable spirometer aligns better with the actual working conditions of the carpenters, as it is the device commonly used for regular health monitoring in a professional setting [10]. This choice reflects the real-life scenario and provides a more realistic assessment of the respiratory health of the workers during their daily activities, contributing to the validity and practicality of the study's findings [10].

**Statistical analysis:** the primary analysis involved calculating the mean differences in spirometric parameters before and after the intervention [5]. Paired t-tests were used to determine the statistical significance of the observed changes. The confidence interval was set at 95% to estimate the precision of the effect size. We used SPSS 26.0 for statistical processing.

**Ethical considerations:** our protocol was submitted to the National GP Ethics Committee, and the recruitment started after obtaining ethical clearance as well as oral consent from each participant. Strict confidentiality measures were implemented to protect their privacy.

## Results

The objective was to assess the impact of FFP3 respiratory mask usage on the respiratory functions of informal sector carpenters in Douala.

**Key results:** improved spirometric parameters: the study found significant improvements in spirometric parameters after the five-month intervention period. Forced vital capacity (FVC) increased from 89.6% to 93.7% (p < 0.000), forced expiratory volume in one second (FEV1) increased from 88.1% to 95.0% (p < 0.000), Tiffeneau index (FEV1/FVC ratio) increased from 82.4% to 84.9% (p < 0.000), and Peak expiratory flow (PEF) increased from 6.7 l/s to 7.9 l/s (p< 0.000).

**Positive impact on lung function:** the regular use of FFP3 masks had a positive effect on the respiratory health of informal sector carpenters. The improvements in spirometric parameters indicated enhanced lung function and reduced airway obstruction.

**Significance of preventive measures:** the study highlights the importance of preventive measures, such as using FFP3 masks, in safeguarding the respiratory well-being of carpenters exposed to occupational hazards. The key results demonstrate that the consistent use of FFP3 respiratory masks significantly enhanced the respiratory functions of informal sector carpenters in Douala. The findings underscore the importance of implementing preventive measures to protect the respiratory health of this specific workforce and highlight the potential benefits of respiratory protective equipment in occupational settings.

**General characteristics of the population:** Table 1 outlines the general characteristics of the population, with the total sample size being n=310. The proportions and corresponding 95% confidence intervals (CIs) are presented for each parameter. In terms of marital status, the majority were married, accounting for 70.7% (95% CI: 69.1- 72.3). Singles represented 29.0% (95% CI: 27.4 - 30.6), while divorced individuals constituted a small percentage of 0.3% (95% CI: 0.28 - 0.32). Regarding educational levels, 0.6% (95% CI: 0.6 - 0.7) had no formal education, 28.3% (95% CI: 26.7 - 29.8) had primary education, 64.5% (95% CI: 62.7 - 66.3) had secondary education, and 6.5% (95% CI: 6.0 - 7.0) had attained a university level education. In terms of years of professional experience, 41.7% (95% CI: 39.8 - 43.6) had less than 10 years, 32.1% (95% CI: 30.4 - 34.8) had 10 to 19 years, and 26.2% (95% CI: 24.7 - 27.1) had 20 years or more of experience. Interestingly, more than a quarter of the carpenters reported having over 20 years of working experience. It's worth noting that only a very small percentage had achieved a university level of education (Table 1).

**Table 1:** general characteristics of the population

Parameter (n=310)	Proportion (%)	IC 95%
**Marital status**		
Married	70.7	69.1 – 72.3
Single	29.0	27.4-30.6
Divorced	0.3	0.28-0.32
**Educational level**		
None	0.6	0.6 – 0.7
Primary	28.3	26.7 – 29.8
Secondary	64.5	62.7 – 66.3
University	6.5	6.0 – 7.0
**Years of professional experience**		
Less than 10 years	41.7	39.8 – 43.6
10 to 19 years	32.1	30.4 – 34.8
20 years and above	26.2	24.7 – 27.1

More than a quarter of the carpenters had more than 20 years of working experience; only a very few had reached university level education

**General anthropological parameters, medical history, and clinical symptoms of the study population:** we adopted a conventional occupational medicine approach in our study population, involving a survey, measurement of anthropological parameters, and examination of medical histories. The average age of our population was 38 years, with a mean body mass index (BMI) at the threshold of overweight. The systolic and diastolic blood pressures were within normal ranges on average. We observed a small proportion of self-reported asthmatics, but a significant percentage of smokers. One-tenth of the population reported drug allergies, and half engaged in physical exercise. One-quarter of the population experienced coughing, and one-fifth had nasal discharge. About one in twenty had ocular irritations or dyspnea, and nearly one-tenth reported chest pain (Table 2).

**Table 2:** anthropological parameters of the study population

Anthropological parameters	Mean	Sd	Min	Max	95% CI
Age (years)	38.0	11.2	18.0	63.0	36.8 – 39.2
Weight (Kg)	75.8	11.8	50.0	130.0	74.5 – 77.1
Height (cm)	171.3	8.3	146.0	200.0	170.4 – 172.2
BMI	26.0	5.2	17.5	90.8	25.4 – 26.6
SBP (mmHg)	127.7	11.2	100.0	180.0	126.5 – 128.9
DBP (mmHg)	82.3	8.9	56.0	110.0	82.2 – 84.2

The examination of anthropological parameters, encompassing age, weight, height, body mass index (BMI), Systolic blood pressure (SBP), and diastolic blood pressure (DBP) within the study population, reveals that the average values are generally within normal ranges, indicating no significant abnormalities

**Spirometric characteristics before the use of masks:** Table 3 provides an overview of spirometric characteristics observed before mask usage. The table includes the mean and standard deviation (sd) values for various parameters, namely FVC (% predicted), FVC (liters), FEV1 (% predicted), FEV1 (liters), FEV1/FVC (% predicted), MEF25-75 (liters per second), and PEF (liters per second). Additionally, the table presents the corresponding minimum and maximum values, as well as the 95% confidence intervals (CIs) for each parameter. The distribution of certain parameters based on percentage ranges is also displayed. For FVC (% predicted), 12.8% measured below 80%, while 87.2% measured equal to or above 80%. In terms of FEV1 (% predicted), 84.1% registered below 80%, and 15.9% measured equal to or above 80%. For FEV1/FVC (% predicted), 8.4% had values below 70%, and 91.6% had values equal to or above 70%. Moreover, the prevalence of obstructive syndrome was found to be 7.8%, and the prevalence of restrictive syndrome was 12.0%, with corresponding 95% CIs of 7.2 - 8.4 and 11.2 - 12.8, respectively (Table 4).

**Table 3:** spirometric characteristics before mask usage

Parameters	Mean	sd	Min	Max	95% CI
FVC (% predicted)	97.6	17.9	25.0	161.0	96.6 – 98.6
FVC (liters)	3.8	0.8	1.0	7.0	2.8 – 4.8
FEV1 (% predicted)	95.9	17.5	30	153	94.9 – 96.9
FEV1 (liters)	3.2	0.7	0.9	5.1	2.2 – 4.2
FEV1/FVC (% predicted)	82.1	8.8	44.1	100.0	81.1 – 83.1
MEF25-75 (liters per second)	3.5	1.3	0.5	7.3	3.4 – 3.6
PEF (liters per second)	7.6	1.8	2.2	14.4	7.4 - 7.7
**Parameters**	**Percentage (%)**				**95% CI**
FVC (% predicted)					
< 80%	12.8				11.9 – 13.7
≧ 80%	87.2				86.3 - 88.1
FEV1 (% predicted)					
< 80%	84.1				83.1 - 85.1
≧80%	15.9				14.7 - 16.9
FEV1/FVC (% predicted)					
< 70%	8.4				7.0- 8.9
≧ 70%	91.6				90.0 - 92.2
Obstructive syndrome	7.8				7.2 - 8.4
Restrictive syndrome	12.0	11.2 - 12.8			

the table provides an overview of spirometric characteristics before mask usage and offers insights into the distribution of these parameters within the studied population; less than a tenth of the carpenters exhibited an obstructive syndrome, while only 12% presented a restrictive syndrome; forced vital capacity (FVC); forced expiratory volume in one second (FEV1); pulsed electric fields (PEF); maximal expiratory flow (MEF)

**Table 4:** other parameters associated with spirometric impairment

Parameter	Mean age	95% CI	P	Mean years of professional experience	95% CI	P
FVC (% predicted) < 80% ≧ 80%	41.3 37.5	41.1- 41.4 37.4-37.6	0.046	15.3 13.4	15.26-15.34 13.36-13.44	0.218
FEV1 (% predicted) < 80% ≧ 80%	37.5 40.6	37.46- 37.54 40.56- 40.64	0.057	13.2 16.0	13.16 -13.24 15.96 - 16.04	0.051
FEV1/FVC (% predicted) < 70% ≧ 70%	46.6 37.2	46.56- 46.64 37.16 -37.24	0.000	17.9 13.2	17.86- 17.94 13.16 -13.24	0.015

The findings indicate that increased exposure to wood dust was associated with worse spirometry parameters; forced vital capacity (FVC); forced expiratory volume in one second (FEV1);

**Parameters associated with spirometric impairment:** Table 4 examines other parameters linked with spirometric impairment. The table compares mean age and mean years of professional experience for specific subgroups based on FVC (% predicted), FEV1 (% predicted), and FEV1/FVC (% predicted). For each subgroup, the mean age is presented with its respective 95% confidence interval (CI) and the associated p-value. In the case of FVC (% predicted), individuals with FVC values below 80% had a mean age of 41.3 years (95% CI: 41.1 - 41.4), while those with FVC values equal to or above 80% had a mean age of 37.5 years (95% CI: 37.4 - 37.6), with a p-value of 0.046. Concerning mean years of professional experience, the respective values were 15.3 years (95% CI: 15.26 - 15.34) for FVC < 80% and 13.4 years (95% CI: 13.36 - 13.44) for FVC >= 80%, with a p-value of 0.218. Similarly, for FEV1 (% predicted), the mean age was 37.5 years (95% CI: 37.46 - 37.54) for FEV1 < 80% and 40.6 years (95% CI: 40.56 - 40.64) for FEV1 >= 80%, with a p-value of 0.057. The mean years of professional experience were 13.2 years (95% CI: 13.16 - 13.24) for FEV1 < 80% and 16.0 years (95% CI: 15.96 - 16.04) for FEV1 >= 80%, with a p-value of 0.051. Regarding FEV1/FVC (% predicted), the mean age for FEV1/FVC < 70% was 46.6 years (95% CI: 46.56 - 46.64), while for FEV1/FVC >= 70% it was 37.2 years (95% CI: 37.16 - 37.24), with a p-value of 0.000. The mean years of professional experience were 17.9 years (95% CI: 17.86 - 17.94) for FEV1/FVC < 70% and 13.2 years (95% CI: 13.16 - 13.24) for FEV1/FVC >= 70%, with a p-value of 0.015 (Table 4).

**Comparative anthropological, clinical, and spirometric characteristics before and after mask usage:** Table 5 presents a comparison of anthropological, clinical, and spirometric characteristics before and after the usage of FFP3 masks. The table outlines the values before and after mask usage, along with the confidence interval (CI) of the difference and the associated p-value (significance level). For BMI, there was negligible change as the values before and after mask usage were very close (24.21 and 24.20 respectively), with a CI of -0.2 to +0.2, resulting in a p-value of 0.91 (NS) indicating no significant difference. In terms of blood pressure, SBP (mmHg) showed a decrease from 125.5 to 121.9 after mask usage, with a CI of 1.2 to 6.0 and a significant p-value of 0.005. Regarding DBP (mmHg), there was minimal change from 80.7 to 80.0 after mask usage, with a CI of -0.4 to +2.1 and a p-value of 0.2 (NS), suggesting no significant difference. Moving to spirometric parameters, significant improvements were observed after mask usage. FVC (% predicted) increased from 89.6 to 93.7, with a CI of -6.0 to -2.3 and a highly significant p-value of 0.000. FVC (liter) increased from 3.7 to 3.9, with a CI of -0.3 to -0.1 and a p-value of 0.000. Similarly, FEV1 (% predicted) increased from 88.1 to 95.0, with a CI of -9.1 to -4.7 and a p-value of 0.000. FEV1 (liter) increased from 3.1 to 3.3, with a CI of -0.3 to -0.2 and a p-value of 0.000. FEV1/FVC (% predicted) increased from 82.4 to 84.9, with a CI of -3.3 to -1.2 and a p-value of 0.000. FEV1/FVC increased from 0.82 to 0.85, with a CI of -0.03 to -0.02 and a p-value of 0.000. MEF25-75 (l/sec) increased from 3.3 to 3.6, with a CI of -0.8 to -0.5 and a p-value of 0.000. PEF (l/sec) increased from 6.7 to 7.9, with a CI of -1.7 to -0.2 and a p-value of 0.000 (Table 5).

**Table 5:** comparative anthropological, clinical, and spirometric characteristics before and after mask usage

Parameter	Before FFP3 mask usage	After FFP3 mask usage	CI of the difference	P (Sig)
BMI	24.21	24.20	- 0.2 ± 0.2	0.91 (NS)
SBP (mmHg)	125.5	121.9	1.2 - 6.0	0.005 (sig)
DBP (mmHg)	80.7	80.0	- 0.4 ± 2.1	0.2 (NS)
FVC (% predicted)	89.6	93.7	- 6.0 – - 2.3	0.000 (Sig)
FVC (litre)	3.7	3.9	- 0.3 – - 0.1	0.000 (Sig)
FEV1 (% predicted)	88.1	95.0	- 9.1 – - 4.7	0.000 (Sig)
FEV1 (litre)	3.1	3.3	- 0.3 – - 0.2	0.000 (Sig)
FEV1/FVC (% predicted)	82.4	84.9	- 3.3 – - 1.2	0.000 (Sig)
FEV1/FVC	0.82	0.85	- 0.03 – - 0.02	0.000 (Sig)
MEF25-75 (l/sec)	3.3	3.6	- 0.8 – - 0.5	0.000 (Sig)
PEF (l/sec)	6.7	7.9	- 1.7 – - 0.2	0.000 (Sig)

Overall, the results demonstrate improvements in spirometric parameters after the carpenters started using FFP3 masks; these improvements are particularly noticeable considering the matched nature of the data, as the carpenters were compared to themselves; forced expiratory volume in one second (FEV1); forced vital capacity (FVC); body mass index (BMI), Systolic blood pressure (SBP), and diastolic blood pressure (DBP); pulsed electric fields (PEF); maximal expiratory flow (MEF); filtering face pieces (FFP3)

## Discussion

The respiratory health of individuals working in the informal carpentry sector is often at risk due to prolonged exposure to wood dust and other airborne pollutants [1,11]. Such exposure has been linked to various respiratory issues, including asthma and other allergic conditions, which can significantly impact the quality of life for these workers. In light of these concerns, the present study aimed to investigate the potential benefits of using FFP3 respiratory protective masks among informal sector carpenters in Douala. This chapter discusses the impact of FFP3 mask usage on the respiratory functions of these carpenters, with a particular focus on Peak Expiratory Flow (PEF), Forced Vital Capacity (FVC) and Tiffeneau Index measurements. These parameters serve as valuable indicators of lung function. The results obtained from a five-month before-after study highlight the positive effects of FFP3 mask intervention, emphasizing the importance of adopting appropriate respiratory protective measures to safeguard the respiratory health of informal sector carpenters in occupational settings. Longitudinal spirometry studies contribute to our understanding of the natural progression and risk factors associated with various respiratory diseases [12,13].

**Forced vital capacity:** the results of our study revealed a significant improvement in forced vital capacity (FVC) among informal sector carpenters in Douala following regular use of FFP3 masks. This increase in FVC indicates enhanced air volume that the subjects can forcefully exhale after a maximal inhalation. Increased FVC suggests improved overall lung function and reduced airway obstruction. These observations are consistent with findings from other similar studies [3,14], reinforcing the idea that wearing respiratory protective masks can play a crucial role in preserving and enhancing the respiratory health of workers exposed to fine particles and harmful aerosols [15].

**Forced expiratory volume in one second (FEV1):** our study also demonstrated a significant improvement in forced expiratory volume in one second (FEV1) among carpenters after the intervention with FFP3 masks. Forced expiratory volume in one second is a key indicator of airway ability to expel air from the lungs during a forced expiration [3,16]. The increase in FEV1 suggests enhanced lung function and reduced bronchial obstruction. These results align with previous research that highlighted the positive impact of wearing respiratory protective masks on the FEV1 of workers exposed to respiratory health hazards in their professional environment [17].

**Tiffeneau index:** the ratio of FEV1 to FVC, is another crucial parameter to assess the respiratory health of individuals. Our results showed a clear improvement in the Tiffeneau index among informal sector carpenters after using 10FFP3 masks. An increase in this index indicates reduced airway obstruction, suggesting an overall improved respiratory function in the subjects [17,18]. Decreased value of Tiffeneau index is common in works exposed to respiratory hazards. These findings are consistent with previous studies, providing additional evidence that regular use of respiratory protective masks can help prevent and alleviate respiratory problems among workers exposed to high respiratory risk factors.

Peak expiratory flow (PEF): peak expiratory flow (PEF) measures the maximum speed at which an individual can exhale air during a forced expiration. Our results demonstrated a significant improvement in PEF among carpenters following the intervention with FFP3 masks. An increase in PEF indicates improved airflow in the airways and suggests enhanced lung function [19]. These results align with other studies that have also highlighted the positive effects of wearing respiratory protective masks on PEF in workers exposed to polluted occupational environments [19]. In a study of Greek furniture workers, the authors found no difference when comparing office workers to exposed workers. They did not conduct a before-after study, but rather compared workers exposed to chemicals. Their sample size was lower in each of the compared groups [20]. The improvement in PEF among carpenters underscores the importance of prevention of respiratory problems through the regular use of suitable respiratory protective masks[3]. On the other hand, the greater the exposure to hazards, the worse the value of the PEF [2,18,21-23]. Overall, our study provides strong evidence that the regular use of FFP3 masks has a positive impact on the respiratory health of informal sector carpenters in Douala. The significant improvements in forced vital capacity (FVC), forced expiratory olume in one second (FEV1), Tiffeneau index, and peak expiratory flow (PEF) indicate enhanced lung function and reduced airway obstruction. These findings are consistent with previous research, highlighting the crucial role of respiratory protective masks in preserving and enhancing the respiratory well-being of workers exposed to fine particles and harmful aerosols [24]. Implementing preventive measures such as mask-wearing and in some instance playing some sport are essential to safeguard the respiratory health of workers in the informal sector and improve overall occupational health outcomes in similar settings [25].

**Generalizability:** while the study's findings provide valuable insights into the impact of FFP3 mask usage on the respiratory functions of informal sector carpenters in Douala, it is crucial to exercise caution when extrapolating these results to other populations or settings. The external validity of the study depends on the context and relevance of the study population and intervention to the target population of interest. For more robust generalizability, replication of the study in diverse populations and settings is necessary.

### Limitation

**Selection bias:** the study's sample consisted of informal sector carpenters in Douala who voluntarily participated. This could introduce selection bias if those who chose to participate were more health-conscious or had better respiratory health than those who opted not to participate (healthy worker effect). As a result, the observed improvements in spirometric parameters might be overestimated if healthier individuals were more likely to be included in the study.

**Compliance bias:** the study relied on self-reported compliance with FFP3 mask usage. Participants may overestimate their adherence to wearing masks consistently, leading to compliance bias. If some participants were less diligent in using masks than reported, the actual impact of FFP3 masks on respiratory functions could be less pronounced than observed.

**Generalizability:** the study was conducted in a specific region (Douala, Cameroon) and among informal sector carpenters. Therefore, the generalizability of the findings to other occupational groups or regions with different working conditions may be limited.

**Confounding factors:** although the study attempted to control for potential confounders (e.g. age, smoking status) in the analysis, there may still be unmeasured or residual confounding that influences the observed results. Factors such as other occupational exposures or pre-existing respiratory conditions could potentially bias the findings.

**Lack of control group:** as a before-after study without a control group, there is no direct comparison to a group not using FFP3 masks. Without a control group, it is challenging to determine if the observed improvements are solely due to the intervention or if other factors could have contributed to the changes in spirometric parameters.

**Duration of intervention:** the five-month intervention period might not be sufficient to capture the long-term impact of FFP3 mask usage on respiratory health. Longer follow-up periods would provide a more comprehensive understanding of the sustained effects of the intervention.

**Self-reported data:** the study relied on self-reported data for certain variables, such as occupational exposure and previous respiratory conditions. Self-reported data may be subject to recall bias and may not be as accurate as data collected through objective measures.

**Sample size:** while efforts were made to achieve an adequate sample size, the study's final sample may still be relatively small, which could limit the precision of the estimated effects.

Considering these limitations, it is essential to interpret the study's findings cautiously. While the results suggest a positive impact of FFP3 mask usage on respiratory functions in informal sector carpenters, the potential biases and uncertainties should be taken into account when drawing conclusions and considering the implications of the study. Further research with larger and more diverse samples, longer follow-up periods, and objective measurements of mask usage would help strengthen the evidence on the effectiveness of FFP3 masks in occupational settings.

**Funding:** the present study on the impact of FFP3 respiratory mask usage on the respiratory functions of informal sector carpenters in Douala was personally funded by the main researcher using personal funds. No external funding was received from any institution or other entity for this research. As the sole founder and main researcher, I personally financed the study, which ensures independence and eliminates any potential conflicts of interest related to funding sources.

## Conclusion

This before-after study conducted among informal sector carpenters in Douala over a five-month period provides compelling evidence for the importance of preventing respiratory problems through regular use of FFP3 respiratory masks. Significant improvements in spirometric parameters, including forced vital capacity (FVC), forced expiratory volume in one second (FEV1), Tiffeneau index, and peak expiratory flow (PEF), were observed among participants who wore the masks during their professional activities. These findings support the beneficial effects of respiratory protective masks on lung function and respiratory health in workers exposed to hazardous occupational environments. Implementing preventive measures, such as wearing FFP3 masks, is crucial to preserve the respiratory health of informal sector carpenters. Increased awareness of occupational risks and collaboration among stakeholders are essential in safeguarding the respiratory well-being of artisans. In conclusion, regular use of FFP3 masks is an effective preventive measure for respiratory problems among informal sector carpenters, reinforcing the need for safe work practices and promoting respiratory health in their profession. These findings contribute to the existing scientific knowledge and strengthen the argument for adopting safe work practices to protect the respiratory health of workers. It is crucial for regulatory authorities, and occupational health authorities to collaborate in promoting the prevention of respiratory problems and enhancing the quality of life for artisans exposed to respiratory hazards in their profession.

### What is known about this topic

- *Negative impact of occupational risks on the respiratory health of informal sector workers;*
- *Potential for improvement in spirometric parameters through preventive measures;*
- *Occupational health disparities in informal sector workers; existing literature has highlighted the disparities in occupational health protection and access to preventive measures among informal sector workers*.

### What this study adds

- *Novel intervention assessment in an understudied population; this study represents a pioneering effort in evaluating the impact of FFP3 mask usage on the spirometric parameters of informal sector workers, specifically carpenters in Douala;*
- *Evidence-based support for respiratory health interventions; with the absence of prior studies investigating the use of FFP3 masks on carpenters' respiratory health, this research provides evidence-based support for the adoption of preventive measures in the informal sector;*
- *Policy implications for respiratory protection in informal sectors: by demonstrating the positive impact of FFP3 mask usage on the respiratory functions of carpenters, this study adds weight to the argument for implementing policies that mandate the use of appropriate respiratory protective equipment in the informal sector*.

## References

[1] Nde Djiele J, Wangata Shadi J, De Brouwer (2015). Formal and informal sector workers care in Cameroon: need for equitable protection approach based on rational assessment of risks and exposures through carpenters respiratory system assessment. Journal of Asthma and Bronchitis.

[2] Mohan M, Aprajita null, Panwar NK (2013). Effect of wood dust on respiratory health status of carpenters. J Clin Diagn Res.

[3] Blanc PD, Iribarren C, Trupin L, Earnest G, Katz PP, Balmes J (2009). Occupational exposures and the risk of COPD: dusty trades revisited. Thorax.

[4] Shamssain MH (1992). Pulmonary function and symptoms in workers exposed to wood dust. Thorax.

[5] Gayatri Vishwakarma (2020). Sample size and power calculation. CBS Publishers and Distributors.

[6] Dupont WD, Plummer WD (1990). Power and sample size calculations: a review and computer program. Control Clin Trials.

[7] Hagstad S, Backman H, Bjerg A, Ekerljung L, Ye X, Hedman L (2015). Prevalence and risk factors of COPD among never-smokers in two areas of Sweden: occupational exposure to gas, dust or fumes is an important risk factor. Respir Med.

[8] Crapo RO, Morris AH, Gardner RM (1981). Reference spirometric values using techniques and equipment that meet ATS recommendations. Am Rev Respir Dis.

[9] Barr RG, Stemple KJ, Mesia-Vela S, Basner RC, Derk SJ, Henneberger PK (2008). Reproducibility and validity of a handheld spirometer. Respir Care.

[10] Redlich CA, Tarlo SM, Hankinson JL, Townsend MC, Eschenbacher WL, Von Essen SG (2014). Official American Thoracic Society Technical Standards: Spirometry in the Occupational Setting. Am J Respir Crit Care Med.

[11] Surber R, Guberan M, Girard JP (1977). Allergies respiratoires aux poussières de bois: Cas cliniques et études épidémiologiques. Revue Française d´Allergologie et d´Immunologie Clinique.

[12] Hannan LM, Dominelli GS, Chen YW, Darlene Reid W, Road J (2014). Systematic review of non-invasive positive pressure ventilation for chronic respiratory failure. Respir Med.

[13] Hnizdo E, Hakobyan A, Fleming JL, Beeckman-Wagner LA (2011). Periodic spirometry in occupational setting: improving quality, accuracy, and precision. J Occup Environ Med.

[14] Holmström M, Wilhelmsson B (1988). Respiratory symptoms and pathophysiological effects of occupational exposure to formaldehyde and wood dust. Scand J Work Environ Health.

[15] Solanki V, Barot K, Chaudhari P (2021). Pulmonary Function Impairment among Stone Cutting Workers in North Gujarat. Int J Health Sci Res 10.

[16] Presti TP, Johnson DC (2023). Improving pulmonary function test interpretation. Eur Respir J.

[17] Al-Neaimi YI, Gomes J, Lloyd OL (2001). Respiratory illnesses and ventilatory function among workers at a cement factory in a rapidly developing country. Occup Med (Lond).

[18] Johncy SS, Ajay KT, Dhanyakumar G, Raj NP, Samuel TV (2011). Dust Exposure and Lung Function Impairment in Construction Workers. JPBS.

[19] Malek F, Ranjbari E, Mirmohammadkhani M, Pahlevan D (2022). Evaluation of pulmonary function among detergent powder factory workers-a cross sectional study in Semnan, Iran. J Occup Med Toxicol.

[20] Paraskevaidou K, Porpodis K, Kontakiotis T, Kioumis I, Spyratos D, Papakosta D (2021). Asthma and rhinitis in Greek furniture workers. J Asthma.

[21] Meo SA (2004). Effects of duration of exposure to wood dust on peak expiratory flow rate among workers in small scale wood industries. Int J Occup Med Environ Health.

[22] Okwari OO, Antai AB, Owu DU, Peters EJ, Osim EE (2005). Lung function status of workers exposed to wood dust in timber markets in Calabar, Nigeria. Afr J Med Med Sci.

[23] Jakubowski M, Abramowska-Guzik A, Szymczak W, Trzcinka-Ochocka M (2004). Influence of long-term occupational exposure to cadmium on lung function tests results. Int J Occup Med Environ Health.

[24] Shadab M, Agrawal DK, Aslam M, Islam N, Ahmad Z (2014). Occupational Health Hazards among Sewage Workers: Oxidative Stress and Deranged Lung Functions. J Clin Diagn Res.

[25] Fatima SS, Rehman R, Saifullah null, Khan Y (2013). Physical activity and its effect on forced expiratory volume. J Pak Med Assoc.

